# Output Stabilization of Wavelength-Swept Laser Based on Closed-Loop Control of Fabry–Pérot Tunable Wavelength Filter for Fiber-Optic Sensors

**DOI:** 10.3390/s22124337

**Published:** 2022-06-08

**Authors:** Byeong Kwon Choi, Soyeon Ahn, Ji Su Kim, Srinivas Pagidi, Min Yong Jeon

**Affiliations:** 1 Department of Physics, College of Natural Sciences, Chungnam National University, 99 Daehak-ro, Yuseong-gu, Daejeon 34134, Korea; bkchoi@o.cnu.ac.kr (B.K.C.); ahnsoyen@o.cnu.ac.kr (S.A.); citrus160@gmail.com (J.S.K.); pagidi.srinivas@gmail.com (S.P.); 2 Institute of Quantum Systems (IQS), Chungnam National University, 99 Daehak-ro, Yuseong-gu, Daejeon 34134, Korea

**Keywords:** wavelength-swept laser stabilization, fiber laser, Fabry–Pérot tunable wavelength filter, fiber-optic sensors, closed-loop control, wavelength stabilization algorithm

## Abstract

The output of a wavelength-swept laser (WSL) based on a fiber Fabry–Pérot tunable filter (FFP-TF) tends to shift the peak wavelength due to external temperature or heat generated by the FFP-TF itself. Therefore, when measuring the output of WSL for a long time, it is very difficult to accurately measure a signal in the temporal domain corresponding to a specific wavelength of the output of the WSL. If the wavelength variation of the WSL output can be predicted through the peak time information of the forward scan or the backward scan from the WSL, the variation of the peak wavelength can be compensated for by adjusting the offset voltage applied to the FFP-TF. This study presents a successful stabilization method for peak wavelength variation in WSLs by adjusting the offset voltage of the FFP-TF with closed-loop control. The closed-loop control is implemented by measuring the deviation in the WSL peak position in the temporal domain using the trigger signal of the function generator. The feedback repetition rate for WSL stabilization was approximately 0.2 s, confirming that the WSL output and the peak position for the fiber Bragg grating (FBG) reflection spectrum were kept constant within ±7 μs at the maximum when the stabilization loop was applied. The standard deviations of WSL output and reflection peak positions were 1.52 μs and 1.59 μs, respectively. The temporal and spectral domains have a linear relationship; the ±7 μs maximum variation of the peak position corresponded to ±0.035 nm of the maximum wavelength variation in the spectral domain. The proposed WSL system can be used as a light source for temperature or strain-dependent sensors as it compensates for the WSL wavelength variation in applications that do not require a fast scanning rate.

## 1. Introduction

A wavelength-swept laser (WSL) varies its wavelength rapidly and continuously across a wide wavelength range and a narrow linewidth [1,2,3,4,5]. It has been mainly used as a light source for optical coherence tomography (OCT) in biophotonics and for dynamic optical fiber sensors because it has a wide scanning range, fast scanning frequency, and narrow linewidth. Lab-built WSLs offer greater flexibility than commercial ones. For example, even if the optical system is a little more complex, it has the advantage of being able to configure it for a desired purpose at a low price [6]. The WSL comprises a filter that continuously changes the wavelength, the gain medium, and the feedback element. Semiconductor optical amplifiers (SOA) or rare-earth-doped optical fibers are mainly used as gain media. A wide gain medium allows operation over a wide scanning range. Recently, a WSL with a scanning range of 150 nm or higher using two SOAs has been reported [4,7,8,9,10]. A wavelength-tunable filter generally uses a polygonal scanning wavelength tunable filter (PSWTF) [1,2,9,10,11] or a fiber Fabry–Pérot tunable filter (FFP-TF) [3,4,5,6,7,8,12,13,14]. Recently, electro-optic and acousto-optic tunable filters have also been reported [15,16,17]; among these, the FFP-TF and PSWTF are the most commonly used wavelength filters. There are advantages and disadvantages to choosing each filter type for the WSL. Although PSWTFs have difficulty in precise optical alignment in free space, they have a linear one-to-one correspondence between the time and wavelength domains, thus requiring less complex signal processing. Conversely, FFP-TFs have thermal instability, but since a WSL can be implemented with all-optical fibers, difficult optical alignment is not required [6,7,8,13,14,18]. Using a high-speed tunable filter, the WSL can operate at a speed of tens of kHz or higher [2,3,18,19]. FFP-TFs can be implemented at a scanning frequency of tens of kHz or more using the Fourier domain mode locking (FDML) method [3,4,5,8,13,14,20]. However, the time and wavelength domains correspond nonlinearly for WSLs based on FFP-TF because the voltage applied to the filter is a sinusoidal function. Therefore, additional signal processing is required, and the oscillation wavelength is not well maintained due to the FFP-TF thermal instability. When measuring wavelength change in dynamic optical fiber sensors using a WSL, if the WSL output wavelength varies due to thermal instability, the sensor sensitivity is affected [3,4,6,14,18]. In particular, monitoring one wavelength component of the WSL in the temporal domain may reveal a thermally induced drift due to this instability. Therefore, when using an FFP-TF-based WSL light source with thermal drift in a sensor that measures wavelength change, wavelength stabilization is required. Measuring the signal reflected from the fiber Bragg grating (FBG) in the temporal domain makes it difficult to distinguish whether it is a signal from the sensor or a signal due to thermal drift. To improve the accuracy and sensitivity of WSL-based sensors, it is necessary to thermally stabilize the WSL by removing the thermal instability of the filter [4,6,19,20]. Recently, a method for stabilizing the peak wavelength of a light source in an FBG-based optical fiber sensor has been reported. J. Sung, et al. have proposed a method of stabilizing a self-synchronization scheme of a WSL-based FBG sensor [21]. Here, they reported the results of improving the sensing performance in the temporal domain with a self-synchronization scheme by applying multiple sensing processing units (SPUs). Commercially stabilized FFP-TF-based WSL creates an error signal to compensate for the thermal instability of the filter, measures it with photodetector (PD), and controls only a specific wavelength in a closed-loop manner [22].

In this paper, we propose a new method for stabilizing the output signal of the WSL by compensating for the FFP-TF thermally induced drift using WSL temporal domain monitoring. The proposed method performs WSL stabilization by applying an offset voltage to the FFP-TF for closed-loop system control.

## 2. Stabilization Method

**Figure 1:**
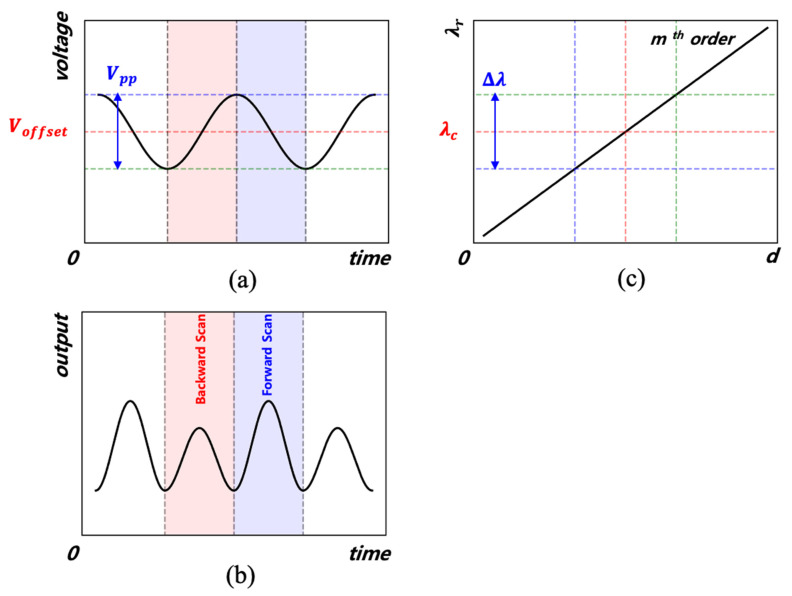
(**a**) Applied voltage of a fiber Fabry–Pérot tunable filter (FFP-TF), (**b**) output of a wavelength-swept laser (WSL) in the temporal domain, and (**c**) the resonant wavelength of a FFP-TF.

When light is vertically incident on the FFP-TF, the resonance wavelength of the *m*-th order is

(1) $$\lambda_{r}=2\;\frac{d}{m\;}\;,$$

where *d* is the distance between the Fabry–Pérot mirrors. When a voltage is applied to the piezoelectric transducer (PZT) inside the FFP-TF, the interval *d* is changed, and thus the transmitted wavelength is changed. When a constant voltage is applied to the PZT, interval *d* is fixed; thus, only a specific wavelength is transmitted. Conversely, when a sinusoidal voltage is applied, the interval *d* changes periodically and thus can be used as a wavelength scanning filter as the transmitted wavelength changes over a specific period. In FFP-TF-based WSLs, when a sinusoidal voltage is applied to the FFP-TF, forward and backward wavelength scans are performed according to the respective voltage variation [3,4,5,6,7,8,13,14,18,19,20]. Here, a forward scan is defined as scanning from a short wavelength to a long wavelength, and a backward scan is the opposite case. Figure 1 shows the change in resonance wavelength *λ_r_* according to the applied FFP-TF voltage and the WSL output. As shown in Figure 1a, when a sinusoidal voltage is applied, the resonance wavelength of the *m*-th order shifts with a range of Δ*λ* centered on the wavelength *λ_c_*, as shown in Figure 1c, where *λ_c_* is the peak wavelength of WSL. The WSL output is shown in Figure 1b; when the voltage applied decreases, the interval *d* increases, and *λ_r_* becomes a longer wavelength. Therefore, the WSL scans twice within one voltage period. However, the interval *d* is affected by both the applied FFP-TF voltage and temperature changes due to the external temperature and heat generated by the applied PZT voltage. Even when a constant voltage is applied to the FFP-TF, the transmitted wavelength varies because of this thermal instability. In general, the output spectrum of WSL is dominated by the gain for each wavelength of SOA. When the area scanned by FFP-TF passes the SOA gain spectrum band, the output of the WSL depending on the SOA gain has one peak per half period of the applied frequency. Although FFP-TF is affected by internal or external thermal perturbation, the peak position of the WSL spectrum maximized is always the same as the wavelength with the largest gain of SOA.

In this study, the peaks of the forward and backward scans were measured by a PD and displayed on an oscilloscope. An electrical signal is directed back to the FFP-TF to adjust the applied offset voltage. By appropriately adjusting the offset and peak-to-peak voltages applied to the FFP-TF, the WSL peak wavelength can be positioned at the point of maximum laser resonator gain. By measuring the WSL intensity in the temporal domain, the largest value can be obtained at the peak wavelength.

**Figure 2:**
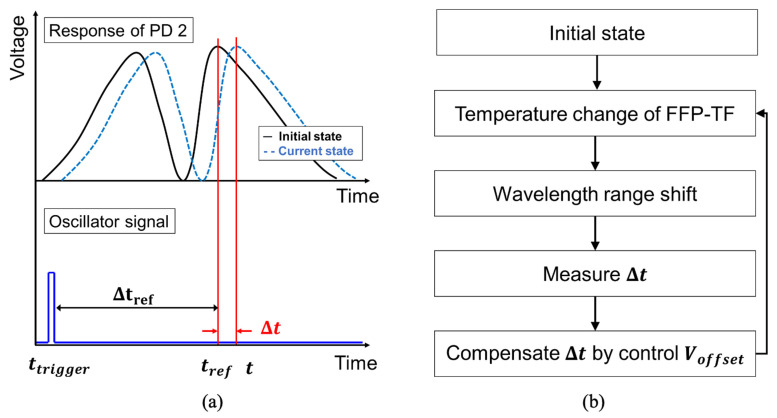
(**a**) The peak position *t_ref_* of the WSL waveform initial state in the temporal domain was measured based on the oscillator trigger signal. The peak wavelength position was shifted to point *t* by altering the external and internal temperatures of the FFP-TF, and (**b**) a schematic of the algorithm based on closed-loop control of the WSL output via the peak position of the forward or backward scan (PD: photodetector).

Figure 2 shows a schematic of the algorithm based on closed-loop control of the WSL output via the peak position of the forward or backward scan [20]. As shown in Figure 2a, the peak position *t_ref_* of the WSL waveform initial state in the temporal domain was measured based on the oscillator trigger signal. The peak wavelength position was shifted to point *t* by altering the external and internal temperatures of the FFP-TF. The time difference Δ*t* = *t* − *t_ref_* caused by the change in the peak position was measured. By adjusting the offset voltage for this change, a feedback voltage was applied to the FFP-TF to correct Δt to close to zero. By repeating this process, as shown in the flowchart in Figure 2b, the peak wavelength was stabilized to *t_ref_*. In general, other factors such as temperature changes of the laser diode (LD) and oscillator can also change the peak point *t_ref_*. However, the LD temperature change is stabilized by the temperature controller, and $\Delta t_{ref}$ does not change because *t_ref_* and *t_trigger_* change simultaneously with the temperature change in the oscillator, as shown in Figure 2a.

## 3. Experiments

**Figure 3:**
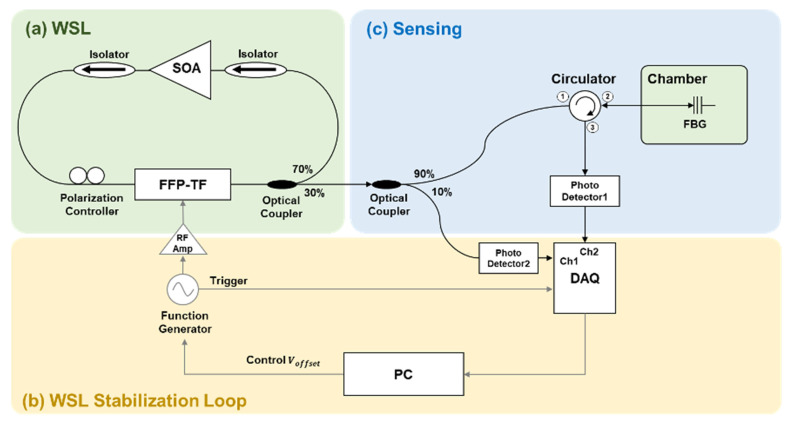
Schematic diagram for stabilizing the wavelength variation in WSL: (**a**) experimental setup of a WSL based on a FFP-TF; (**b**) structure of the closed-loop control; and (**c**) experimental setup for measuring the temperature change signal by placing the FBG in a temperature chamber. (WSL: wavelength-swept laser; SOA: semiconductor optical amplifier; FFP-TF: fiber Fabry–Pérot tunable filter; DAQ: data acquisition board; PC: personal computer; FBG: fiber Bragg grating).

The FFP-TF-based WSL was stabilized by applying a sinusoidal voltage and measuring the waveform in the temporal domain. Figure 3 shows the experimental procedure for stabilizing the thermally induced wavelength variation in the WSL. Figure 3a shows the experimental setup of the WSL based on a FFP-TF. An SOA in the 1550 nm band was used as a gain medium, and optical isolators were placed on either side of the SOA to propagate light in one direction. As the SOA has a polarization dependence, the WSL output was optimized by appropriate adjustment using a polarization controller. The WSL output was obtained using a 30:70 output coupler. Ten percent of the WSL output was measured using a 14-bit data acquisition (DAQ) board (high speed digitizer 5122, National Instrument) sampling at 100 M samples/s through a PD 2. Figure 3b shows the structure of the closed-loop control system for WSL stabilization. The signal received through the DAQ is fed back to the FFP-TF through the RF amp, and the WSL output stabilization is performed by adjusting the applied offset voltage, V_offset_, using a personal computer (PC). For the experiments, a 5 V_pp_ voltage and 100 Hz scanning frequency were applied to the FFP-TF. V_offset_ was adjusted such that the WSL peak wavelength was similar to the center wavelength of the SOA amplified spontaneous emission (ASE) spectrum. Figure 3c shows the experimental setup for confirming the performance of the stabilized WSL. The sensor signal for temperature change was measured by placing an FBG in a temperature chamber. After 90% of the WSL output was incident on the FBG through the optical circulator, the reflected wavelength was measured by the PD and an optical spectrum analyzer (OSA). The FBG was installed in the chamber maintained at 20 °C; if the chamber temperature is constant, the reflected wavelength from the FBG will also be constant. When the reflected signal from the FBG is measured in the temporal domain without applying the WSL stabilization loop, only the thermally induced wavelength drift can be measured.

## 4. Experimental Results

**Figure 4:**
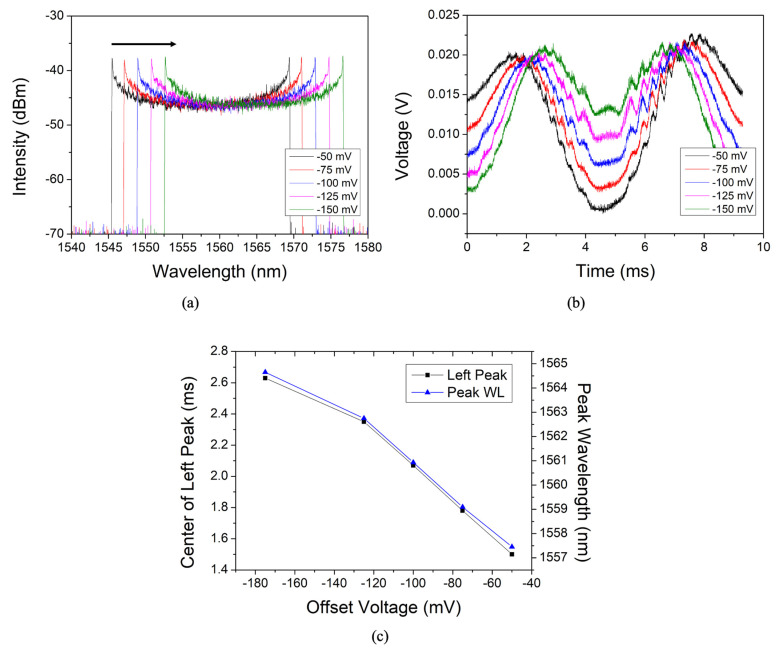
(**a**) WSL outputs in the spectral domain; (**b**) WSL outputs in the temporal domain; and (**c**) the variation of the center wavelength of WSL output in the spectral domain (right axis) and the variation of the forward scan peak in the temporal domain (left axis) with respect to V_offset_.

Figure 4a,b show the output results of the WSL measured in the spectral and temporal domains, respectively, according to the V_offset_ applied to the FFP-TF. Figure 4a shows that the measured spectral bandwidth in the spectral domain was shifted to the longer wavelength with respect to V_offset_. As previously stated, the WSL has a linear one-to-one correspondence between the spectral and temporal domains [3,4,23,24,25]. Figure 4b shows that in the corresponding temporal domain, the distance between the two peaks of the forward and backward scans decreases, and the central overlapping portion between the two scans increases. The peaks of the forward scan (left) shift to the right in the temporal domain and overlap each other. This is a phenomenon that occurs as the wavelength band of WSL moves to a longer wavelength. When this overlap between scans increases, the wavelength component at the overlapping portion cannot be accurately measured. Figure 4c shows the variation of the center wavelength of WSL output in the spectral domain (right axis) and the variation of the forward scan peak in the temporal domain (left axis) with respect to V_offset_. The variation of the central wavelength and the peak position show almost the same trend.

When measuring the thermally induced variation in the peak wavelength in the temporal domain, achieving good measurement accuracy is extremely important. Accuracy is affected by the WSL tuning range, scanning frequency, and bandwidth of the measuring instruments, including the PD and DAQ. The WSL scanning range is determined by the peak-to-peak voltage V_pp_ applied to the FFP-TF. If V_pp_ is slightly increased within the free spectral range (FSR) while the scanning frequency is kept constant, the range of the corresponding wavelengths for the same time interval slightly increases [26], and thus, the measurement accuracy decreases. Similarly, if the bandwidth of a measurement device is constant, increasing the WSL scanning frequency will cause a wider range of wavelengths to be measured at the same time interval, thus reducing the measurement accuracy. In these experiments, a peak-to-peak voltage of 5 V_pp_ and a scanning frequency of 100 Hz were applied to the FFP-TF to easily observe the wide wavelength band of WSL and the change of the scan range by the applied V_pp_. The applied V_pp_ and scan frequency depend on the bandwidth of the PD and DAQ used in the experiments. The PD and DAQ have bandwidths of 100 MHz and 50 MHz, respectively; therefore, the maximum measurable bandwidth is limited to 50 MHz. If the scan frequency applied to the FFP-TF is increased, the bandwidth of the PD and DAQ should be increased. The accuracy in compensating for the thermally induced wavelength change is determined by the FSR per voltage of the FFP-TF and the resolution of the offset voltage change by the oscillator. The oscillator used in this experiment was the Agilent 33120A with an offset voltage resolution of 0.01 mV, a gain of 10 from the RF amplifier, and a smallest controllable resolution of 0.1 mV. In addition, the FSR of our FFP-TF was 210 nm and the full tuning voltage was approximately 18 V. Therefore, when 0.1 mV was adjusted, the FFP-TF center wavelength changed by approximately 1.16 pm.

**Figure 5:**
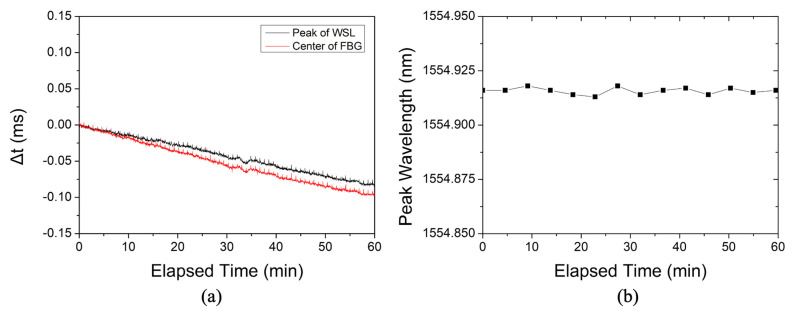
When the WSL stabilization loop is not applied, (**a**) variations of peak position in the temporal domain (black line: peak position of the WSL, red line: reflected signal from the FBG); and (**b**) variation of the peak wavelength reflected from the FBG in the spectral domain.

Figure 5 shows the variation of peak position of the reflected signal from the FBG when the WSL stabilization loop is not applied (i.e., temperature compensation is not applied). Figure 5a shows the variation in the peak positions of the WSL output and FBG reflection signals in the temporal domain for 1 h. The drift Δ*t* confirmed a change of −0.10 ms when measured for 1 h. This does not mean a maximum wavelength drift, and depending on the external environment, a larger Δ*t* may occur or a smaller Δ*t* may occur. The position of the reflected signal peak from the FBG changes similarly to the peak wavelength position for the WSL output. Figure 5b shows the result of measuring the variation in the peak wavelength reflected from the FBG in the spectral domain for 1 h. As predicted, it can be seen that the peak wavelength reflected from the FBG in the wavelength domain is kept constant, but there is a drift in the FBG reflection peak in the time domain.

**Figure 6:**
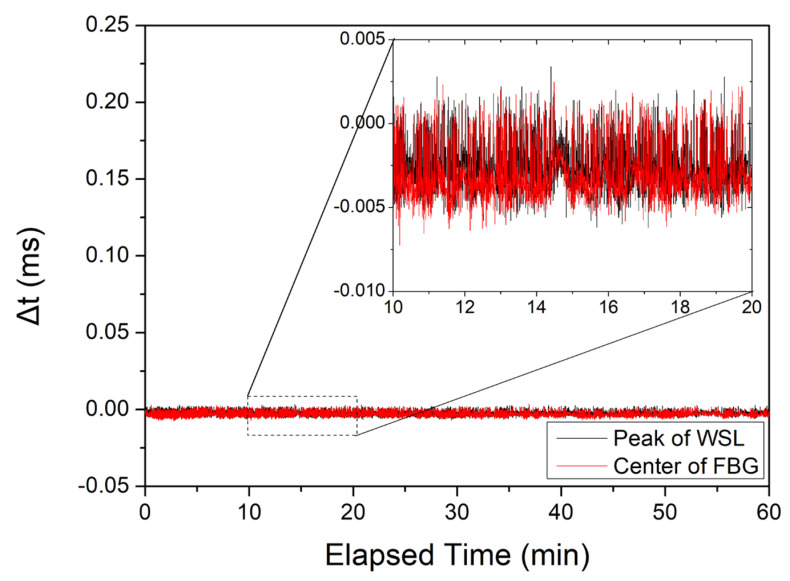
Range of peak position, Δ*t* of WSL (red line) and FBG (black line) in the temporal domain when a WSL stabilization closed-loop control system is applied.

Figure 6 shows the variation of peak positions of the reflected signal from the FBG when the WSL stabilization control system is applied (i.e., FFP-TF temperature compensation is applied), where the feedback repetition rate for WSL stabilization was approximately 0.2 s, and Gaussian fitting was used to find the peak position using the WSL forward scan. The results confirm that the WSL output and the peak position for the FBG reflection spectrum were kept constant within ±7 μs at the maximum when the stabilization loop was applied. The relationship between the change amount Δ*t* in the temporal domain and the change amount *Δλ_c_* in the spectral domain is determined by the wavelength scanning range Δ*λ* and the scanning frequency *f*. In this experiment, the wavelength scanning range and the scanning frequency were approximately 25 nm and 100 Hz, respectively. For the forward scan, a wavelength range of 25 nm was scanned for 5 ms. The standard deviations of the WSL output and the reflected peak position were 1.52 μs and 1.59 μs, respectively. Therefore, as the peak positions of the forward and backward scans are located in the linear part of the applied sinusoidal voltage, and assuming that the temporal and spectral domains have a linear relationship, the ±7 μs maximum variation of the peak position corresponds to ±0.035 nm in the spectral domain.

## 5. Conclusions

This study, to the best of our knowledge, has successfully compensated for the variation in the WSL peak wavelength by using closed-loop control to adjust the offset voltage of the FFP-TF. Closed-loop feedback was implemented by measuring the WSL peak position variation in the temporal domain using the trigger signal of the function generator. The stabilization algorithm was implemented using the feedback signal to apply an offset voltage to the FFP-TF to compensate for the WSL peak wavelength variation. When the stabilization algorithm was applied, the WSL output was stabilized with a maximum ±0.035 nm peak wavelength range. The accuracy of the peak variation measurement is limited according to the bandwidth of the output photodetector when the WSL scanning frequency is increased to the 10–100 kHz range. However, we believe WSLs can be used as a light source for temperature or strain sensors because the proposed method can simply compensate for the WSL wavelength variation in applications that do not require high scanning frequencies [27,28].

## Data Availability

Data available on request from the authors.

## References

[1] Yun S.H., Richardson D.J., Kim B.Y. (1998). Interrogation of fiber grating sensor arrays with a wavelength-swept fiber laser. Opt. Lett..

[2] Oh W.Y., Yun S.H., Tearney G.J., Bouma B.E. (2005). 115 kHz tuning repetition rate ultrahigh-speed wavelength-swept semiconductor laser. Opt. Lett..

[3] Huber R., Wojtkowski M., Fujimoto J.G. (2006). Fourier domain mode locking (FDML): A new laser operating regime and applications for optical coherence tomography. Opt. Express.

[4] Jeon M.Y., Zhang J., Wang Q., Chen Z. (2008). High-speed and wide bandwidth Fourier domain mode-locked wavelength swept laser with multiple SOAs. Opt. Express.

[5] Adler D.C., Wieser W., Trepanier F., Schmitt J.M., Huber R. (2011). Extended coherence length Fourier domain mode locked lasers at 1310 nm. Opt. Express.

[6] Kassani S.H., Villiger M., Uribe-Patarroyo N., Jun C., Khazaeinezhad R., Lippok N., Bouma B.E. (2017). Extended bandwidth wavelength swept laser source for high resolution optical frequency domain imaging. Opt. Express.

[7] Zhang J., Jing J., Wang P., Chen Z. (2011). Polarization-maintaining buffered Fourier domain mode-locked swept source for optical coherence tomography. Opt. Lett..

[8] Lee G.H., Ahn S., Gene J.W., Jeon M.Y. (2021). 1.1-µm Band Extended Wide-Bandwidth Wavelength-Swept Laser Based on Polygonal Scanning Wavelength Filter. Sensors.

[9] Cui L., Bashir A., Javaid A., Lee S., Ha J. (2022). Broadband wavelength swept laser with bidirectional photonic resonance for high resolution optical coherence tomography. IEEE Photonics J..

[10] Cao J., Wang P., Zhang Y., Shi G., Wu B., Zhang S., Liu Y. (2017). Methods to improve the performance of the swept source at 1.0 μm based on a polygon scanner. Photonics Res..

[11] Cao Y., Wang L., Lu Z., Wang G., Wang X., Ran Y., Feng X., Guan B. (2019). High-speed refractive index sensing system based on Fourier domain mode locked laser. Opt. Express.

[12] Lee B.C., Jung E.-J., Kim C.-S., Jeon M.Y. (2010). Dynamic and static strain fiber Bragg grating sensor interrogation with a 1.3 μm Fourier domain mode-locked wavelength-swept laser. Meas. Sci. Technol..

[13] Park J., Kwon Y.S., Ko M.O., Jeon M.Y. (2017). Dynamic fiber Bragg grating strain sensor interrogation with real-time measurement. Opt. Fiber Technol..

[14] Chen M., Li H., Chen R. (2014). Swept laser source based on acousto-optic tunable filter. Proc. SPIE.

[15] Han G.-H., Cho S.-W., Park N.S., Kim C.-S. (2016). Electro-optic swept source based on AOTF for wavenumber-linear interferometric sensing and imaging. Fibers.

[16] Park N.S., Chun S.K., Han G.-H., Kim C.-S. (2017). Acousto-optic-based wavelength-comb-swept laser for extended displacement measurements. Sensors.

[17] Jirauschek C., Huber R. (2015). Wavelength shifting of intra-cavity photons: Adiabatic wavelength tuning in rapidly wavelength-swept lasers. Biomed. Opt. Express.

[18] Jun C., Villiger M., Oh W.-Y., Bouma B.E. (2014). All-fiber wavelength swept ring laser based on Fabry-Perot filter for optical frequency domain imaging. Opt. Express.

[19] Kolb J.P., Pfeiffer T., Eibl M., Hakert H., Huber R. (2018). High-resolution retinal swept source optical coherence tomography with an ultra-wideband Fourier-domain mode-locked laser at MHz A-scan rates. Biomed. Opt. Express.

[20] Eigenwilling C.M., Biedermann B.R., Palte G., Huber R. (2008). K-space linear Fourier domain mode locked laser and applications for optical coherence tomography. Opt. Exp..

[21] Sung J.-Y., Chen J.-K., Liaw S.-K., Kishikawa H., Goto N. (2020). Fiber Bragg grating sensing system with wavelength-swept laser distribution and self-synchronization. Opt. Exp..

[22] https://lunainc.com/product/si155-optical-sensing-instrument.

[23] Kwon Y.S., Ko M.O., Jung M.S., Park I.G., Kim N., Han S.P., Ryu H.C., Park K.H., Jeon M.Y. (2013). Dynamic sensor interrogation using wavelength-swept laser with a polygon-scanner-based wavelength filter. Sensors.

[24] Ko M.O., Kim S.-J., Kim J.-H., Lee B.W., Jeon M.Y. (2014). Dynamic measurement for electric field sensor based on wavelength-swept laser. Opt. Express.

[25] Ko M.O., Kim S.-J., Kim J.-H., Jeon M.Y. (2018). In situ observation of dynamic pitch jumps of in-planar cholesteric liquid crystal layers based on wavelength-swept laser. Opt. Express.

[26] Jeon M.Y., Zhang J., Chen Z. (2008). Characterization of Fourier domain mode-locked wavelength swept laser for optical coherence tomography imaging. Opt. Express.

[27] Feng Z., Cheng Y., Chen M., Yuan L., Hong D., Li L. (2022). Temperature-compensated multi-point strain sensing based on cascaded FBG and optical FMCW interferometry. Sensors.

[28] Yuan Q., Wang Z., Song L., Lu Z., Hu D., Qin J., Yang T. (2019). A fast linearly wavelength step-swept light source based on recirculating frequency shifter and its application to FBG sensor interrogation. Sensors.

